# Simulated patients in medical education – a survey on the current status in Germany, Austria and Switzerland

**DOI:** 10.3205/zma001235

**Published:** 2019-05-16

**Authors:** Michael Sommer, Angelika Hiroko Fritz, Christian Thrien, Angelika Kursch, Tim Peters

**Affiliations:** 1 Dresden University of Technology, Medical Interprofessional Training Center, Dresden, Germany; 2 University of Duisburg-Essen, Faculty of Medicine, Simulation Patient Program, Essen, Germany; 3 University of Cologne, Cologne Interprofessional Skills Lab and Simulation Center KISS, Cologne, Germany; 4 Hannover Medical School, Research and Teaching Unit Med. Psychology, Hannover, Germany; 5 hsg Bochum, Department für Pflegewissenschaft, Bochum, Germany

**Keywords:** Medical education, medical studies, SP program, simulated patients

## Abstract

**Objective: **In German-speaking countries (Germany, Austria, Switzerland), simulated patients (SPs) have been a fixture for years and are used in teaching and examinations. As part of ongoing methodological standardization efforts and to support current and future faculty and curriculum developments, this exploratory study systematically investigates how and under what framework and conditions SPs are currently used in German-speaking countries.

**Methodology:** The online questionnaire developed in cooperation with the Committee for Simulated Patients of the Society for Medical Education comprises 58 questions covering the organization and administration, size and design of the SP pool, general conditions and minimum standards for the assignments of the SPs. All medical faculties from Germany, Austria and German-speaking Switzerland were invited to participate in the survey and a descriptive data analysis was performed.

**Results: **38 responses from 45 faculties were included in the evaluation of the survey (response rate: 84.4%). Most SP programs are affiliated with the Office of the Dean of Studies and skills labs or training centers and funded by faculty resources. Both the working hours in the SP programs and the qualifications of the employees vary extensively. The same applies to the number and average age of the employed SPs. On average each faculty uses 1,290 SP hours per year (min=45, max=6,500). The majority of SPs are used in a teaching environment, together with lecturers. At all sites, SPs provide feedback to students. This is always based on a uniform standard. All SPs receive training, which predominantly focuses on playing their role and giving feedback.

**Discussion: **There are a variety of SP programs in German-speaking countries. While there are a few clear similarities (for example, feedback from SPs), many organizational and methodological aspects are handled differently. Although this allows innovation and flexibility, it also weakens the didactic SP method in its standardization and thus in the comparability of quality. A certain degree of standardization and high methodical quality is of great importance, especially in scientific and faculty internal discussions and with a view to the use of SPs in high-stakes examinations which must be improved in the future.

## Zusammenfassung

**Zielsetzung:** Im deutschsprachigen Raum (Deutschland, Österreich, Schweiz) sind Simulationspatientinnen und Simulationspatienten (SPs) seit Jahren eine feste Größe und werden in Lehre und Prüfungen eingesetzt. Im Rahmen laufender methodischer Standardisierungsbestrebungen und um aktuelle wie künftige Fakultäts- und Curriculumentwicklungen zu unterstützen, arbeitet die explorative Studie systematisch auf, wie und unter welchen Rahmenbedingungen SPs im deutschsprachigen Raum aktuell eingesetzt werden.

**Methodik: **Der in Kooperation mit dem Ausschuss Simulationspatienten der Gesellschaft für Medizinische Ausbildung entwickelte Onlinefragebogen umfasst 58 Fragen zu den Kategorien Organisation und Administration, Größe und Ausgestaltung des SP-Pools, Rahmenbedingungen und Mindeststandards bei den Einsätzen der SPs. Alle Medizinischen Fakultäten aus Deutschland, Österreich und der deutschsprachigen Schweiz wurden zur Teilnahme an der Umfrage eingeladen. Die Daten wurden anschließend deskriptiv ausgewertet.

**Ergebnisse: **38 Antworten von 45 Fakultäten flossen in die Auswertung der Umfrage ein (Rücklaufquote: 84,4%). Die meisten SP-Programme sind an den Studiendekanaten sowie Skills Labs bzw. Trainingszentren angegliedert und durch Haushaltsmittel der Fakultäten finanziert. Sowohl der Stellenumfang in den SP-Programmen als auch die Qualifikation der dortigen Mitarbeitenden fallen sehr unterschiedlich aus. Gleiches gilt für die Anzahl und den Altersdurchschnitt der eingesetzten SPs. Im Mittel werden an jeder Fakultät 1290 SP-Einsatzstunden pro Jahr geleistet (min.=45; max.=6500). Die Mehrheit der SPs wird zusammen mit Dozierenden in Lehrveranstaltungen eingesetzt. An allen Standorten geben SPs den Studierenden Feedback. Diesem liegt immer ein einheitlicher Standard zugrunde. Alle SPs erhalten Schulungen, die überwiegend Rollen- und Feedbacktrainings sind.

**Diskussion: **Es existiert eine große gelebte Vielfalt bei den SP-Programmen im deutschsprachigen Raum. Während es wenige klare Gemeinsamkeiten gibt (z.B. Feedback der SPs), werden viele organisatorische und methodische Aspekte unterschiedlich gehandhabt. Dies erlaubt zwar Innovation und Flexibilität, schwächt aber gleichzeitig die didaktische Methode SP in ihrer Standardisierung und damit in der Vergleichbarkeit der Qualität. Gerade bei wissenschaftlichen und fakultätsinternen Diskussionen und mit Blick auf den Einsatz von SPs in high-stakes-Prüfungen ist eine gewisse Standardisierung und eine methodisch hohe Qualität allerdings von großer Bedeutung, an der in Zukunft gearbeitet werden muss.

## 1. Introduction

Simulated patients (SPs) are an established component of education, training and further education in national and international health care. The specially trained (lay) actors credibly [1] take on the role of patients and other roles in the health care system to facilitate teaching and examining scenarios in medical education [2]. First developed by Howard Barrows in the 1960s for the teaching of neurology [3], the method is now used worldwide across the whole range of health professionals and beyond [4]. One of the reasons for this wide adoption in medical education is due to the methodological advantages of SPs, which are widely described in the literature [5]. They include, among other things, flexible availability, repeatability and predictability of presentations, the ability to perform reliable examinations and the ethical acceptability of using SP even for difficult scenarios [6], [7], [8], [9].

In German-speaking countries SPs have occasionally been used since the 80s but they were not used frequently until after the year 2000 [5], [10], [11], [12]. Since then, they have been firmly established as a didactic element in teaching and examinations in many areas and the number of standardized patient programs in the D-A-CH region (Germany, Austria, Switzerland) has steadily increased. The planned or already implemented integration into the final medical examinations will further increase the importance of simulation patients and, with them, also the questions of institutional anchoring and actual methodological implementation at the various locations [13], [14].

As far back as 2004, Fröhmel et al. investigated the integration of simulated patients in medical education [10]. At that time, 13 out of 30 responding faculties reported working with SPs. Since the survey only referred to Germany and the data is already more than ten years old, the question arose as to how the existing SP programs in the entire D-A-CH region are currently positioned and how exactly they work with the SP method. It was therefore decided to assess the current state of SP programs through an exploratory study. This study was also an essential basis for the development of the position paper “Minimum Standards and Development Perspectives in the Use of Simulated patients” [15].

## 2. Methods

A digital survey was conducted in 2016 to collect the data. A standardized questionnaire was used, analogous to the study by Fröhmel et al. [10]. This was developed by the authors in a multi-stage process and is based on the guidelines of Döring and Bortz [16]. As a pre-test, the survey instrument was discussed critically in open workshops with SP experts at the GMA conference 2015 in Leipzig as well as at the International Skills-Lab Symposium 2016 in Essen and the face validity was checked. Then incomprehensible or ambiguous language was eliminated. The final questionnaire consisted of 58 individual questions on the topics:

- Organization of the SP program
- Design of the SP pool
- Existing framework conditions
- Minimum standards when using SPs

The questionnaire included both multiple-choice and open-ended questions to enable a more precise description of the diversity in the various faculties. The pre-test suggested that the questionnaire should be anonymous and ignore the question of faculty affiliation. This aspect as well as human resource issues excluded a qualitative survey (such as telephone interviews). 

The questionnaire was sent to all 45 medical faculties in German-speaking countries on April 27, 2016. The link to the questionnaire was sent by email either directly to the known SP programs or to the Office of the Dean of Studies or other central teaching bodies. The recipients of the survey were asked in the cover email to complete only one questionnaire per SP program (see attachment 1 ). The survey was completed in August 2016. The survey was carried out using the online tool EvaSys. The data was then processed descriptively. For the evaluation of some open questions, a clustering procedure was chosen to categorize the heterogeneous answers. For this purpose, the answers were viewed separately by all authors, categories were formed and the results were finally brought together in a consensus process. 

## 3. Results

In total, the questionnaire was filled in 38 times. It cannot be ruled out that there were multiple responses from a single faculty. However, examination of the data revealed that no dataset resembles another across multiple items in a conspicuous manner. Assuming that none of the 45 medical faculties submitted multiple responses during the survey phase, this equates to a response rate of 84%. Most answers come from employees of German faculties (32). There were five responses from Switzerland and one from Austria. All respondents indicated that their faculty has at least one SP program. 31.6% of respondents said that there are several SP programs at their faculty. On average, the responding faculties use 1,290 SP hours per year (min=45, max=6,500).

### 3.1. Organization of SP programs

**Figure 1:**
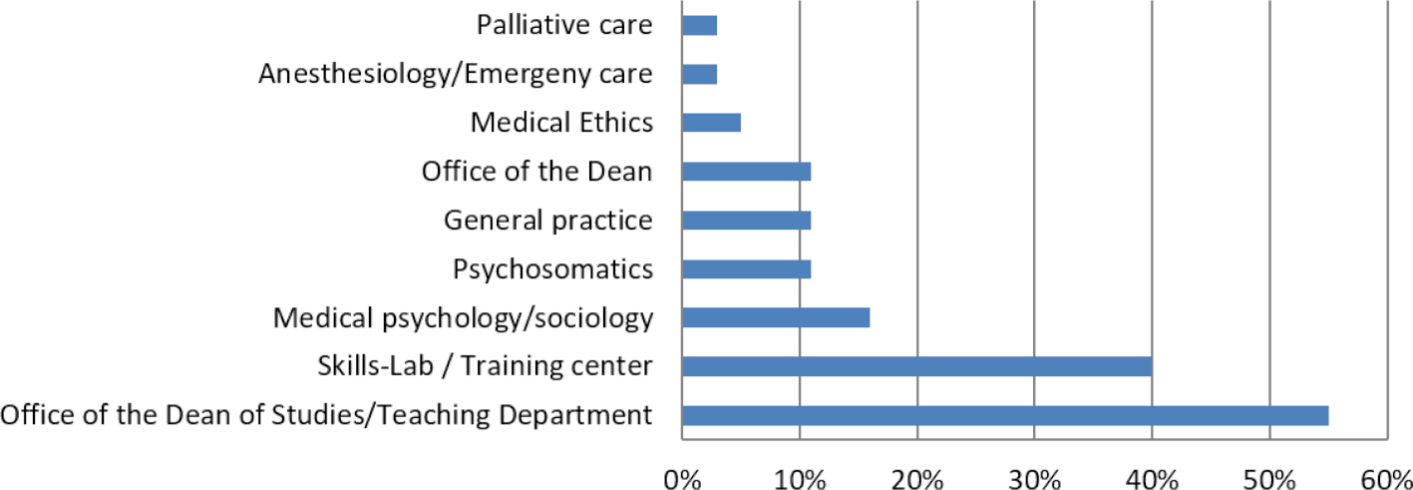
Organizational units to which the SP programs are affiliated

The SP programs have different institutional affiliations. Organizationally, most of them belong to the Office of the Dean of Studies/Teaching Department and/or to the skills lab/training center (see figure 1 (Fig. 1)). In some cases, SP programs are linked to several structural units.

25 respondents answered the question as to the source of the SP program funding at their site. Most frequently, the faculty’s budget was used as the funding base (see table 1 (Tab. 1)). Because of many similarities in terminology (e.g. “study fees” and “study grants”), there is the possibility that different terms refer to the same concept. Multiple answers were possible. 

Almost every year since 2002, a new SP program has been established in German-speaking countries. Most of the respondents’ SP programs were established between 2004 and 2009 (22). One faculty has been working with SPs since 1980.

The number of students at the faculties varies considerably. The “smallest” responding faculty enrolls 160 students per year, the largest 660 students (MW=316).

81% of respondents said that the SP program is well established in their faculty. However, only 61% of respondents said that there are people responsible for the SP program at their location who were explicitly employed for this purpose. 

In total, 30 respondents provided information on the weekly hourly input into their SP program. The values range from 0.5 to 124 hours per week for the SP program (SD=29.9 hrs). The average number of working hours per SP program is 38.1 hours. 

According to the respondents, the SP programs mainly employ psychologists (22%) and physicians (20%). The remainder is made up of employees from a nursing background, theater pedagogy, speech and linguistics, archeology, the acting professions and tutors from health professions. 18% of respondents said that their SP program employs people for coordination and team assistance. 65% of the respondents stated that the employees of the SP program receive special training for their job at their location. These are mainly SP coaching courses (see table 2 (Tab. 2)). 

In addition, the employees of the SP programs have very heterogeneous other qualifications, for example in the areas of: Drama, psychodrama, naturopaths, systemic counseling, psychotherapy, Master of Medical Education, communication training, supervision, clowning.

According to the respondents, an average of 3.5 student assistants work on each SP program (min=0, max=40). The total number of hours invested by student assistants per SP program ranges from 0 to 44 hours per week. Most student assistants work on data entry (34%), tutoring (32%) and production of teaching materials (29%). Amongst others, they also perform the following tasks: Organization/coordination, designing classes, billing and public relations.

#### 3.2. Information on the SPs used 

47% of respondents said that the SPs from their SP program are also used in other disciplines (such as dentistry, nursing, psychology, economics) and 71% of respondents said that the SPs are also used in the continuing education of physicians or other health professionals.

The SP programs of the responding faculties employ on average 61 SPs (min=8, max=260). The age range of the employed SPs ranges from 6 to 89 years. 71% of respondents said their SPs are contracted.

#### 3.3. Framework and conditions of SP assignments

At the faculties surveyed SPs are used in almost all departments with patient contact. Most commonly in psychosomatic and internal medicine (both 58%), followed by medical sociology and psychology (50%) and psychiatry and surgery (both 47%).

Most respondents (95%) indicated that the SPs are deployed at their location alongside teaching staff, with 55% of respondents stating their SPs are also used alongside student tutors. It was reported that in 13% of the assignments, the SPs were alone with the students. Multiple answers were possible. In line with this, 8.1% of respondents stated that the SPs at their faculty are also engaged as teaching staff.

At all faculties which responded, SPs are used in simulations as patients with a focus on communication. Other types of assignments are for example communication as a relative (79%), production of (educational) films and physical examinations with role script (each 71%).

In teaching situations, the SPs at all faculties which responded give feedback; in exam situations, however, only up to 21%. All interviewees indicated that SPs’ feedback is always based on common standards (for example PID principle: perception – impact – desire).

61% of respondents stated that their SP program has clearly formulated policies for the protection of SPs. The protective measures are summarized in table 3 (Tab. 3).

If compensation is paid to the SPs, it ranges between 5-75 Euros (6-80 CHF) per hour. The various SP programs differ considerably in how they stagger their remuneration rates, mostly according to the following criteria:

- Difficulty of the role
- Number of roles played by one SP
- Length of affiliation to the SP program
- Use with/without feedback
- Dialog/physical examination
- Journey distance of the SPs
- Type of assignment (training/examination/teaching/educational film production/photo shoot)

11% of respondents said that there are SPs in their faculty who are volunteers. Five respondents stated that their SP program reimburses travel expenses.

The administration of the SPs is software-based in 63% of the responding faculties, with mainly Microsoft Access and Excel being used as well as SimPat and Item Management System (IMS).

In order to render authentic simulations, 70% of the responding SP programs use opaque windows and 54% special simulation rooms (especially medical practice rooms, treatment or patient rooms). Two respondents indicated that an ICU was constructed for their simulations. One faculty uses a simulated dental practice. 58% of respondents said that they use makeup (for example realistic accident representation).

#### 3.4. Minimum standards for SP assignments

All 38 respondents indicated that their faculty used a standard procedure to recruit SPs. Different priorities are set at the faculties (see table 4 (Tab. 4)). Multiple answers were possible.

All 38 respondents said the SPs working at their medical school receive training. This is mainly in the form of role and feedback training (see table 5 (Tab. 5)). 

17 of the 38 respondents stated that they use tools to measure the quality of SP assignments and SP feedback (see table 6 (Tab. 6)). Multiple answers may lead to overlaps in this section.

## 4. Discussion

The aim of the survey was to obtain an overview of the current state of the framework conditions and working principles of SP programs in German-speaking countries. Of the 45 medical schools contacted, we received 38 replies. The high response rate results in good explanatory power. Also, analysis of the data sets suggests that each data set represents a different SP program, even if multiple responses were received from one faculty (multiple SP programs at a faculty).

In summary, all responding medical schools have at least one SP program and SPs are used in teaching (with a focus on communication and the physical examination) and student assessment. In addition, the high number of hours per year show that the use of simulated patients is no exception but is a well-established high-performing teaching component in medical degree courses. In addition, SPs at half of the responding medical faculties are also used in teaching contexts outside of human medicine (for example, dentistry or health and nursing), demonstrating the didactic value of the SP method. 

Nevertheless, the SP method is conducted or implemented very differently at each location. This is clear from the number of SP assignments, the way they are embedded into teaching and thus also the size of the SP programs, which vary greatly. The affiliation of SP programs within their institutions (mainly in Office of the Dean of Studies and skills labs of the faculties) and the fact that many disciplines with patient contact employ SPs suggests that intra-faculty development of the curricula towards more practical and simulated training and examinations has been established and that SP programs are seen as an integral part of education. This is also reflected in the fact that the SP programs are largely funded through the faculties’ budgets. 

At the same time, the very different working conditions of the permanent employees in SP programs indicate that the workplace is very heterogeneous and that in some cases sufficient institutional support is not always provided. This is clearly visible in the areas of working hours, activities and qualification measures. For example, all respondents refer to quality assurance tools, for example training for the SPs and continuing education for staff, however, only 61% of the respondents said that people responsible for the standardized patient program were hired explicitly, which makes focusing and long-term professional quality assurance more difficult. The collected data suggest that the SP programs at many medical faculties are coordinated by employees who actually have a job in a different area.

It was observed that training of SPs (for example in the areas of presentation and feedback) was ubiquitous in all of these programs and that certain elements, such as the feedback given by SPs, have become standard in teaching situations. In exams with SPs (for example OSCE), only one in four responding faculties use feedback-giving SPs. Presumably, the rigidly timed examination procedures prevent incorporating SP feedback or there is disagreement over how to handle the impact of feedback on the exam objectivity.

Most SPs receive compensation for their work – even if it varies greatly. It should be seen critically that 11% of responding faculties employ SPs on a voluntary basis. From the perspective of the authors, this is not adequate considering the time cost and the emotional investment of SPs nor the quality requirements for role presentation and feedback. The same applies to SPs who receive a fee that is below the statutory minimum wage. 

At the same time, the exact design of the SP programs as well as the standards or specifications used for aspects of SP assignments are very heterogeneous, especially in the area of minimum standards and quality assurance. The locations seem to have independently developed criteria and processes, an examination of which would be beyond the scope of this exploratory study. 

This, according to the authors, shows both the strength and weakness of the SP programs in the D-A-CH region: There is a great variety of SP methods practiced in medical teaching. Depending on the location and the individual needs, this allows flexible deployment and opens the door to innovations and further developments. While this may be acceptable with regards to organizational matters, it creates difficulties in the qualification of the SPs and the methodological framework for their assignments, unless minimum standards are met. As much as various other forms of teaching and examinations (such as OSCE) have attempted to enhance and refine their methodology in recent years, there seems to be a rather heterogeneous approach to professionalism when working with SPs. To put it bluntly: If each site can unilaterally implement the SP method, what remains of the method? And what is the scientific foundation which it requires, especially when it is used in testing or even final examinations? Based on these questions, the authors of this exploratory study dealt with the minimum requirements and development perspectives of SP programs and published them in a position paper [15].

Future work will have to show how on the one hand to maintain a variety of practice and on the other hand, how the SP method can be qualitatively enhanced at the same time in order to make it fit for use in high-stakes examinations. 

## 5. Limitations

To capture the diversity of the reality of SP programs, an exploratory study was conducted with a correspondingly high number of open questions. Nevertheless, the quantitative data is of interest, for example on the qualifications of the people responsible for the SPs or forms of financing. In some cases, evaluation was difficult because free-form answers were not clearly classifiable. For this reason, the figures generated from it are only informative to a certain extent.

We received only one answer from Austria, so this cannot be seen as representative for the country. In Switzerland, only the German-speaking faculties were included in the survey, which calls transferability to the whole of Switzerland into question. Of the faculties which did not respond, it cannot be ascertained whether they do or do not have SP programs.

The feedback from colleagues at a workshop on the survey at the annual meeting of the Society for Medical Education in Bern in 2016 showed that the questions “Are there clearly formulated measures to protect SPs?” and “What measures are used to protect SPs? (for example: SPs perform a maximum of 3 times consecutively per day)” were understood very differently. On the one hand, there were differences in the understanding of the term “measures” and, on the other hand, there was disagreement as to the precise meaning of “clearly formulated”. The same applies to the question about the software used to administer the SPs. Since 37% of the respondents stated that they did not use any software, the authors suspect that the question regarding software was interpreted differently and in some cases was assumed to refer to specialized SP software. 

Further research should take into account that in the D-A-CH region SP programs have also been implemented at other educational institutions (such as nursing or physiotherapy training centers). This survey only focused on the SP programs at medical faculties.

## Acknowledgements

Our sincere thanks to all individuals and locations who participated in the survey. We would also like to thank the members of the “Simulated Patients” committee for their constructive participation in the questionnaire. 

## Notes

The “Simulated Patients” committee of the Society for Medical Education (GMA) was renamed the “Simulated Persons” committee in February 2019. This text uses the old name because the article was developed prior to the name change.

## Competing interests

The authors declare that they have no competing interests. 

## Supplementary Material

Simulated Patients in Medical Education – a survey on the current status in Germany, Austria and Switzerland

## Figures and Tables

**Table 1:**
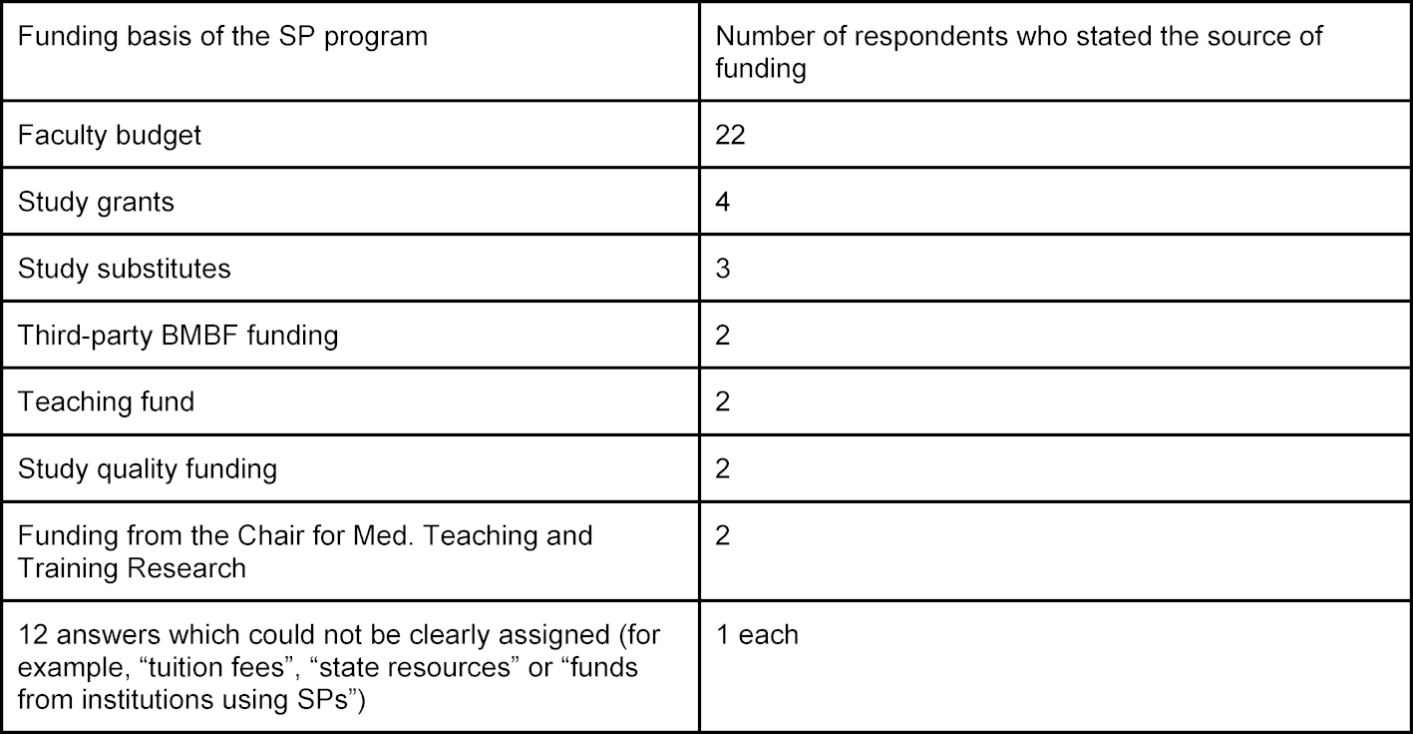
Funding basis of SP programs

**Table 2:**
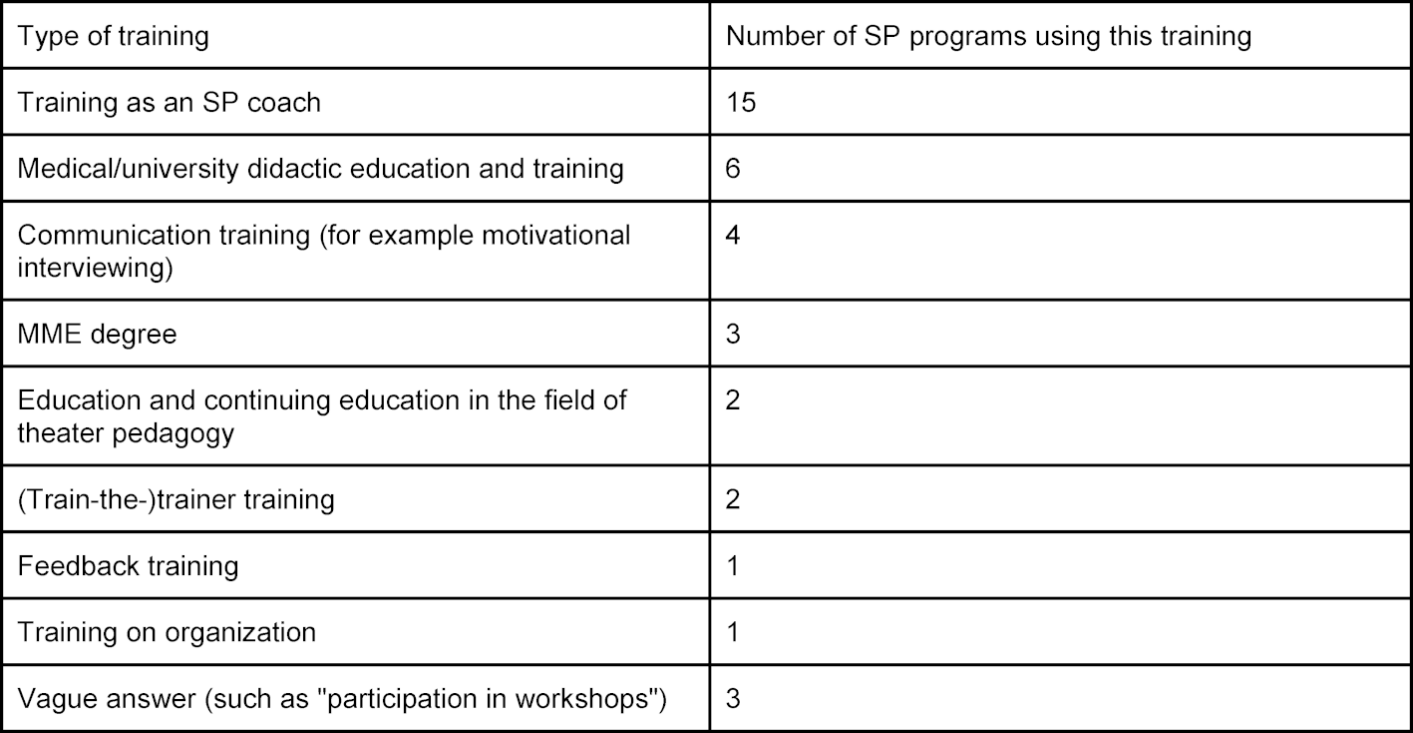
Continuing education provided to employees of SP programs

**Table 3:**
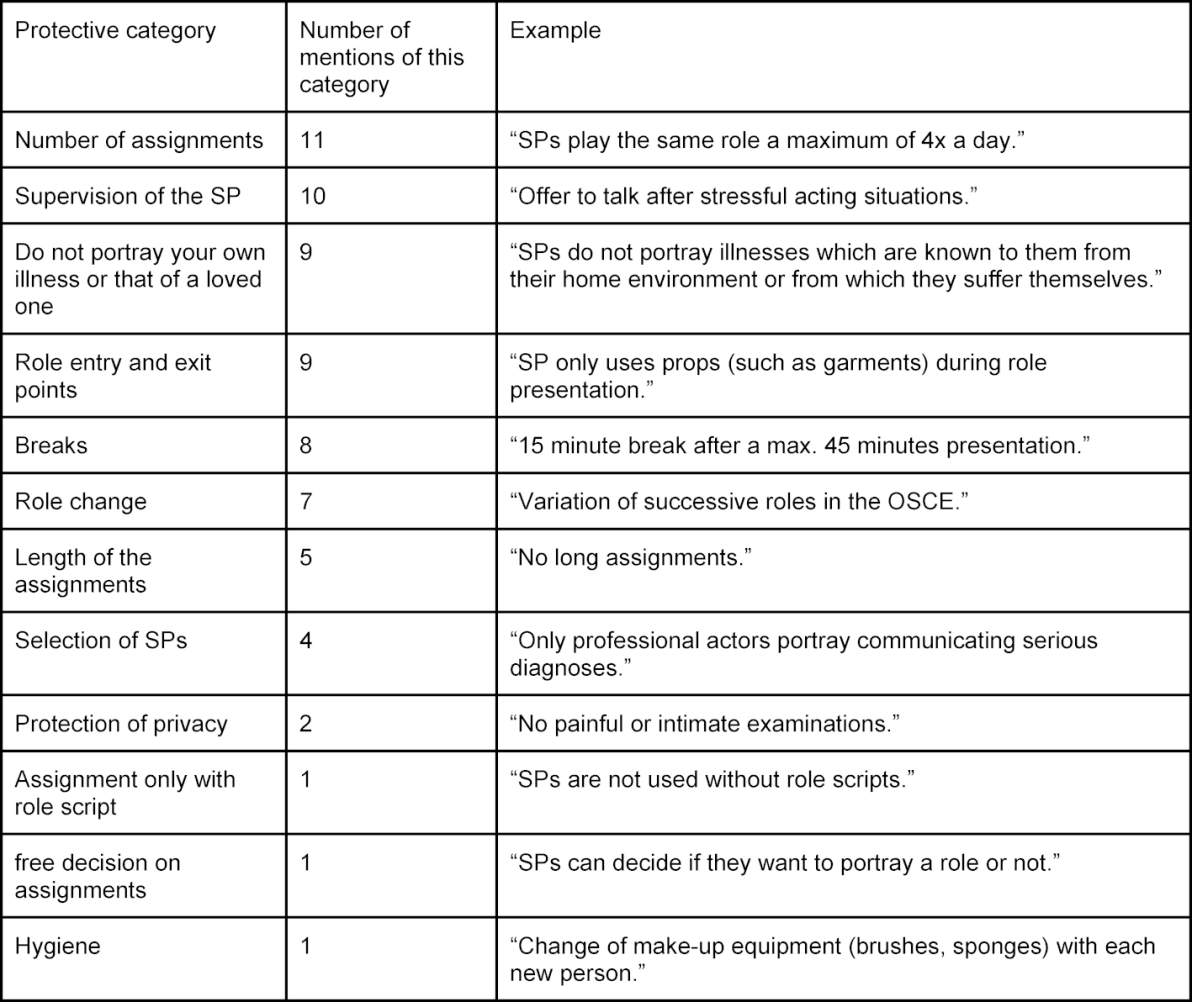
Protective Measures for SPs

**Table 4:**
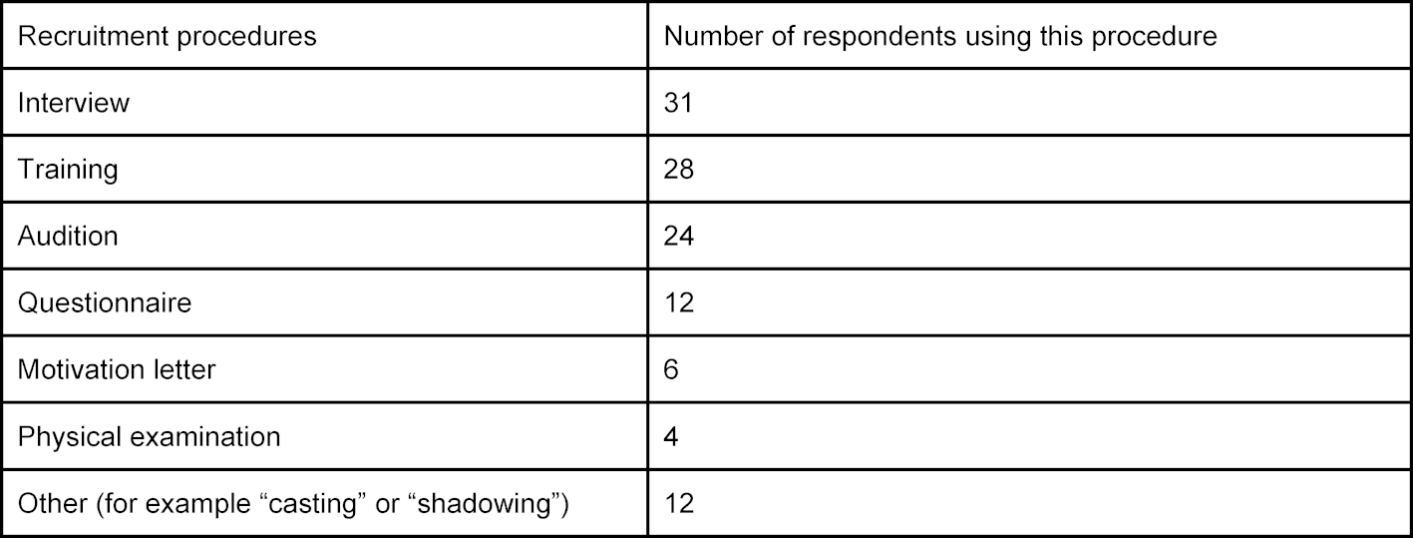
Recruitment Procedure for SPs

**Table 5:**
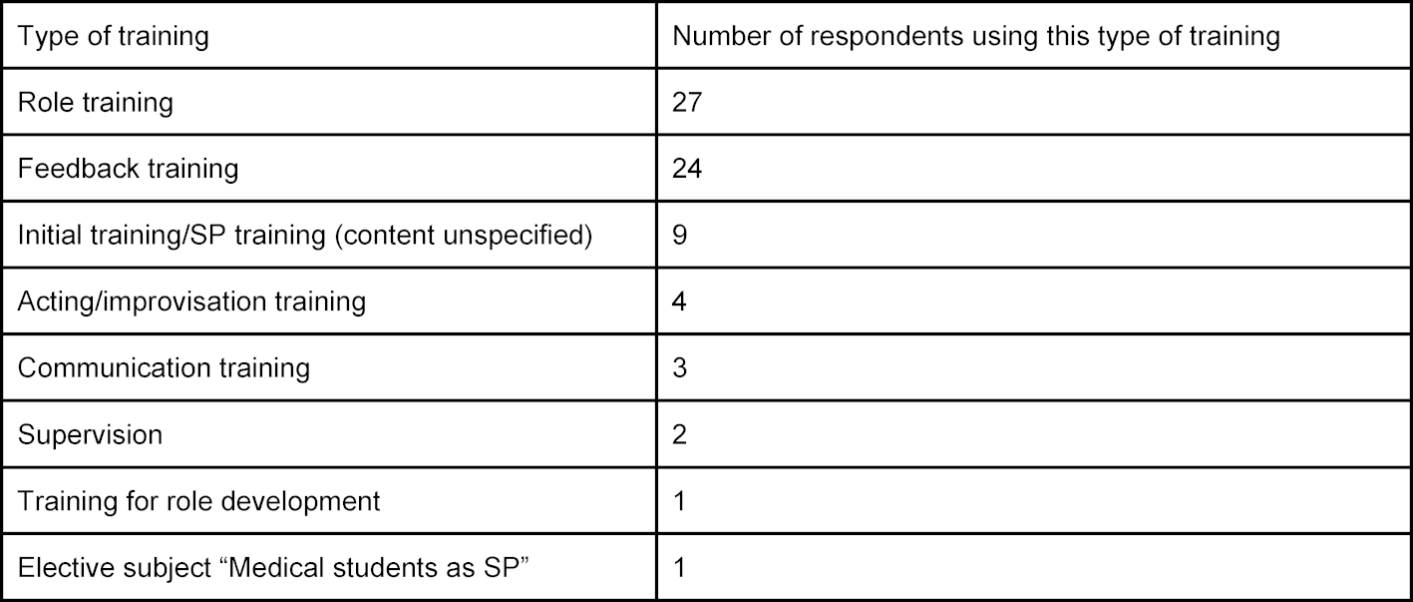
Training for SPs

**Table 6:**
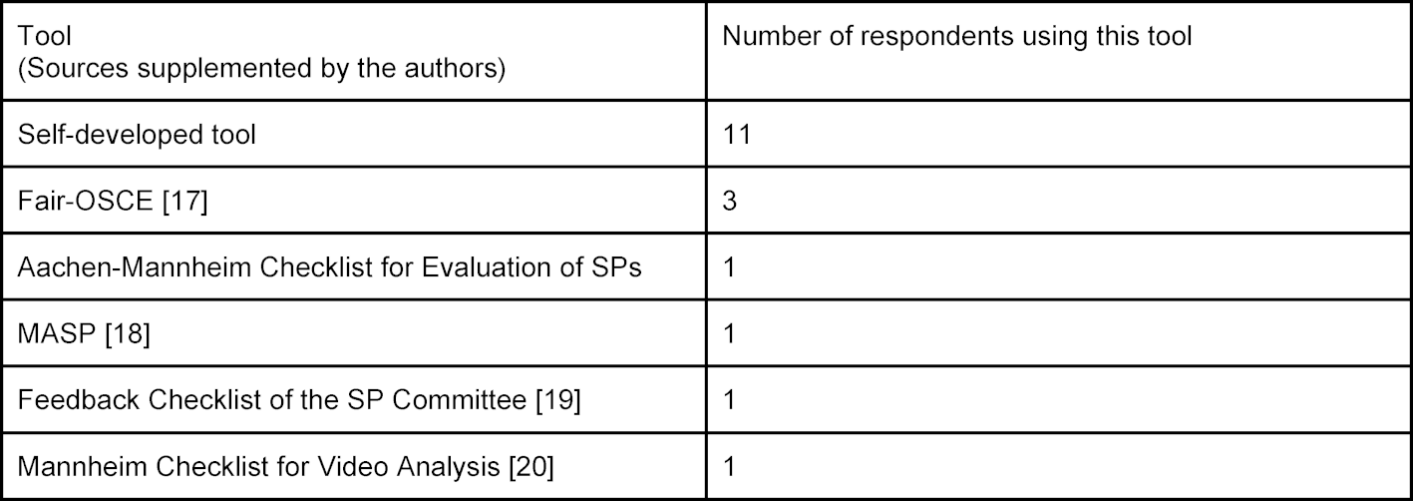
Tools Used for Quality Assurance of Feedback from SPs
