# Supplementary material for: Simulated patients in medical education – a survey on the current status in Germany, Austria and Switzerland
Source: GMS J Med Educ. 2019 May 16;36(3):Doc27. doi: 10.3205/zma001235 (PMC6545614; doi:10.3205/zma001235)
Supplement: Simulated Patients in Medical Education – a survey on the current status in Germany, Austria and Switzerland [file JME-36-3-27-s-001.pdf]

Survey cover email

Dear colleagues,

Enclosed you will find a link to a survey which aims to investigate the current state of SP programs in German-speaking countries for a position paper of the “Simulated Patients” committee of the Society for Medical Education (GMA). You will benefit from participation in the survey by becoming aware of your own framework conditions and by receiving feedback on the results of the survey as a whole.

Please complete the questionnaire at the link below by 05.13.2016 or forward this mail to the staff responsible for simulated patients at your faculty.

The survey can be started here:

https://eval.med.tu-dresden.de/evasys/online.php

TAN: SP-Programm

Depending on the situation, completing the survey may take between 20-40 minutes. For orientation and your own documentation, we also attach the questionnaire in PDF-format. Please only use the online questionnaire for submitting your answers.

Please fill out the online questionnaire only once per SP program. If there are several SP programs at your location, we ask you to forward the survey to any SP programs that may not have been contacted by us.

The background to our survey:

Two years ago, at the GMA meeting in Hamburg, the “Simulated Patients” committee (https://gesellschaft-medizinische-ausbildung.org/ausschuesse/simulationspatienten.html) set up a project group that would like to publish a position paper on “Minimum standards for working with simulated patients”. The position paper should help us to scientifically secure the quality of the method used with simulated patients, analogous to similar exam concepts and teaching methods and to publicly document this. In addition to this form of quality assurance and professionalism, such a paper can and should also provide arguments for future discussions on the use of SPs within the faculties and their funding. Therefore, the position paper should be a handy resource for all of us in facilitating our work in a variety of ways.

This survey is not a research project with a specific research question but merely serves to generate a rough overview of the current state of affairs in order to outline this in the position paper. Your data will also be treated confidentially and will not be published on-site. No personal data will be collected and the results will only be used anonymously. Individual sections may be left empty if necessary, but we would ask for an explanation in such cases.

If you have any questions or encounter ambiguities, we are at your disposal (Tim Peters, Michael Sommer and Angelika Hiroko Fritz).

The Position Paper Team thanks you for your cooperation and wishes you all a nice weekend.

Survey of SP programs in German-speaking countries

1. Introduction

Dear colleagues,
in this questionnaire we hope to collect data on standardized patient programs in German-speaking countries.
Please complete the questionnaire only for your program and if necessary forward the questionnaire to other SP programs at your location.

2. Questions about the organization of the SP program

No.	Question	Answer
2.1	In which country is your SP program based?	□ Germany<br>□ Austria<br>□ Switzerland
2.2	Are there multiple SP programs at your medical faculty?	□ Yes<br>□ No
2.3	How many SP programs are there at your medical faculty?	
2.4	How is your SP program affiliated within the institution?	□ Office of the Dean of Studies/Teaching Department<br>□ Skills-Lab/Training Center<br>□ Medical Psychology/Medical Sociology<br>□ Psychosomatics<br>□ Medical Ethics<br>□ Other: (see 2.5.)
2.5	To which other institutions is your SP program affiliated?	
2.6	How is the SP program financed at your location? (for example by the faculty, teaching fund, study grant etc. ...)	
2.7	Since when has your medical faculty been working with SPs? (Year)	
2.8	Since when did your independent SP program exist? (Year)	
2.9	Is your SP program temporary or firmly established?	□ Temporary project<br>□ Firmly established
2.10	Do you have any people responsible for SP at your location who have been specifically hired for the SP program?	□ Yes<br>□ No

2.11	What are the (estimated) working hours in your SP program (ignoring tutors, SPs and student assistants)? For employees without a permanent contract, please state the estimated workload.<br>Answer format:<br>Profession of employee 1, working hours per week. Profession of employee 2, working hours per week....	
2.12	What other qualifications do the responsible employees of the SP program have? (for example specialist doctor, psychotherapist, supervisor)	
2.13	Did/do the employees receive special training for their work?	□ Yes<br>□ No
2.14	What special training did the employees receive? (for example SP coach training, training in motivational interviewing)	
2.15	How many student assistants work in the SP program on average? (Enter number)	
2.16	What is the overall number of hours done by student assistants per week on average? (Enter number)	
2.17	Which tasks are carried out by student assistants?	□ Teaching (Tutoring)<br>□ Designing classes<br>□ Creation of teaching materials<br>□ Data entry<br>□ Other: (see 2.18)
2.18	What other tasks are carried out by student assistants?	
2.19	How many students are enrolled every year for human medicine at your university?	

3. Questions about your SP

No.	Question	Answer
3.1	How big is the SP pool currently?	
3.2	How old is the youngest SP you use?	
3.3	How old is the oldest SP you use?	
3.4	Do you also use SPs from your SP program in other fields (other than human medicine)? (for example dentistry, health professions, education, economics ...)	□ Yes<br>□ No
3.5	In which other areas do you use SPs?	

3.6	Are SPs used in medical continuing education (specialization of doctors or continuing education in health professions)?	□ Yes<br>□ No
3.7	How many paid SP hours per year were completed in your SP program (real contacts in teaching and examinations, ignoring SP training)?	

4. Questions about the framework conditions when using SPs

No.	Question	Answer
4.1	Which departments in the human medicine degree program at your faculty work with SPs?	□ Anesthesia and intensive care<br>□ Occupational and social medicine<br>□ Ophthalmology<br>□ Surgery<br>□ Dermatology<br>□ History/Medical ethics<br>□ Gynecology<br>□ Otorhinolaryngology<br>□ Internal Medicine<br>□ Medical sociology and psychology<br>□ Neurology<br>□ Orthopedics<br>□ Pediatrics<br>□ Pharmacology and toxicology<br>□ Psychiatry<br>□ Psychosomatics<br>□ Forensic medicine<br>□ Urology<br>□ Other: (see 4.2)
4.2	Which other departments in the human medicine degree program at your faculty work with SPs?	
4.3	How are SPs used in teaching students? (Multiple answers possible)	□ Alongside lecturers<br>□ Alongside student tutors<br>□ Alone with students

4.4	What types of SP assignments do you have? (Multiple answers possible)	□ Communication (as patient)<br>□ Communication (as a relative)<br>□ Communication (in an interprofessional setting)<br>□ Communication (in other function, which see 4.5)<br>□ Physical examination (with role script)<br>□ Physical examination (without role script)<br>□ Physical examination with model assignment (hybrid ward)<br>□ Production of films (such as educational films, training films, promotional films etc.)
4.5	In what other functions are SPs used?	
4.6	Do the SPs give feedback?	□ Yes<br>□ No
4.7	Where do the SPs give feedback?	□ In teaching<br>□ In exams
4.8	Is the feedback based on uniform standards and is a specific method used for guidance?	□ Yes<br>□ No
4.9	Which standards or methods is the feedback based on? (for example PID principle, understanding-oriented feedback, sandwich-method)	
4.10	Do the SPs take on lecturer functions in teaching situations?	□ Yes<br>□ No
4.11	In which courses/training wards do SPs assume lecturer functions?	
4.12	How are the SPs remunerated? (Description of the remuneration, please be as detailed as possible)	
4.13	Do you use SPs on a voluntary basis?	□ Yes<br>□ No
4.14	Do the SPs receive a contract?	□ Yes<br>□ No
4.15	Is administration of the SPs software-based?	□ Yes<br>□ No
4.16	What special software is used for SP administration? (for example databases such as IMS or Access)	

4.17	Are you researching with SPs? (are SPs involved in research in any form whatsoever?)	□ Yes<br>□ No
4.18	Are there other medical departments doing research with SPs?	□ Yes<br>□ No
4.19	Which medical departments apart from you are researching with SPs?	
4.2	Do you use video feedback in simulations?	□ Yes<br>□ No
4.21	Do you use rooms with opaque windows in simulations with SPs?	□ Yes<br>□ No
4.22	Do you use special simulation rooms (for example hospital, consultation room, intensive care unit, simulation arena etc.)?	□ Yes<br>□ No
4.23	Which special simulation rooms do you use?	
4.24	Do you use make-up for simulations (for example RAR = Realistic Accident Representation)?	□ Yes<br>□ No
4.25	Are there clearly formulated measures to protect SPs?	□ Yes<br>□ No
4.26	What measures do you use to protect SPs? (For example: SPs play a role a maximum of 3 times in a row during the day)	

5. Questions about the minimum standards when using SPs

No.	Question	Answer
5.1	Is there a standard procedure for the employment of SPs?	□ Interview<br>□ Questionnaire<br>□ Audition<br>□ Physical examination<br>□ Motivation letter<br>□ Training<br>□ Other (see 5.2)
5.2	What other procedures do you use to employ SPs?	
5.3	Do your SPs receive training?	□ Yes<br>□ No

5.4	What training do your SPs receive? Answer format:<br>Training 1, average length of training, frequency per year, participation optional vs. mandatory<br>Training 2, average length of training, frequency per year, participation optional vs. mandatory...	
5.5	Do you regularly use specific instruments to measure the quality of SP assignments and feedback (such as MASP)?	□ Yes<br>□ No
5.6	What instruments do you use to measure the quality of SP assignments and feedback?	
